# Bacterial Composition, Genotoxicity, and Cytotoxicity of Fecal Samples from Individuals Consuming Omnivorous or Vegetarian Diets

**DOI:** 10.3389/fmicb.2017.00300

**Published:** 2017-02-28

**Authors:** Ermanno Federici, Roberta Prete, Camilla Lazzi, Nicoletta Pellegrini, Massimo Moretti, Aldo Corsetti, Giovanni Cenci

**Affiliations:** ^1^Laboratory of Microbiology, Department of Chemistry, Biology and Biotechnology, University of PerugiaPerugia, Italy; ^2^Faculty of BioScience and Technology for Food, Agriculture and Environment, University of TeramoTeramo, Italy; ^3^Department of Food Science, University of ParmaParma, Italy; ^4^Department of Pharmaceutical Sciences, University of PerugiaPerugia, Italy

**Keywords:** vegetarian diet, omnivores, viable fecal bacteria, fecal water, genotoxicity, cytotoxicity

## Abstract

This study analyzes the composition of viable fecal bacteria and gut toxicology biomarkers of 29 healthy volunteers, who followed omnivorous, lacto-ovo-vegetarian, or vegan diets. In particular, the research was focused on the prevalence of some representative viable bacteria from the four dominant phyla (Firmicutes, Bacteroidetes, Proteobacteria, Actinobacteria) commonly present in human feces, in order to evaluate the relationship between microorganisms selected by the habitual dietary patterns and the potential risk due to fecal water (FW) genotoxicity and cytotoxicity, considered as biomarkers for cancer risk and protective food activity. The relative differences of viable bacteria among dietary groups were generally not statistically significant. However, compared to omnivores, lacto-ovo-vegetarians showed low levels of total anaerobes. Otherwise, vegans showed total anaerobes counts similar to those of omnivores, but with lower number of bifidobacteria and the highest levels of bacteria from the *Bacteroides–Prevotella* genera. FW genotoxicity of lacto-ovo-vegetarians resulted significantly lower either in relation to that of omnivores and vegans. Lacto-ovo-vegetarians also showed the lowest levels of cytotoxicity, while the highest were found for vegans. These results highlighted that lacto-ovo-vegetarian diet was particularly effective in a favorable modulation of microbial activity, thus contributing to a significant reduction of the genotoxic and cytotoxic risk in the gut.

## Introduction

Knowledge concerning chronic intestinal diseases, such as irritable bowel syndrome, inflammatory bowel disease, Crohn’s disease, and colorectal cancer (CRC), emphasizes their genetic, immunological, metabolic multifactorial etiology and indicates that lifestyle and dietary habits may also have an important role in their origin and progression (Hope et al., 2005; Mai et al., 2009). The implications between diet and health are highlighted by epidemiological and experimental studies (Claesson et al., 2012; Adebola et al., 2013; Conlon and Bird, 2015). A high intake of animal fats and proteins typical of western diets, especially when associated with a low fiber intake, is considered a potential risk factor in the etiology of colon cancer (Pearson et al., 2009). On the contrary, lacto-vegetarian diets are believed to have a protective role in CRC incidence (Sabate, 2003; Pettersson et al., 2008). Besides that, long-term diets affect the dynamics of gut microbial communities, which play an important role in host physiology and, consequently, in health or disease status (Zimmer et al., 2012; Scott et al., 2013; Matijasic et al., 2014). A well-balanced gut microbiota, beside promoting gastrointestinal systemic functions, acts as a barrier against pathogenic microorganisms, influences the immune system, controls epithelial cell proliferation and differentiation, produces essential metabolites and bioactive components (Mai et al., 2009; Mariat et al., 2009; Zhu et al., 2011). It can thus be envisaged that in condition of equilibrium the microbiota could limit the risk of carcinogenicity (Shah, 2007; Fotiadis et al., 2008). The beneficial outcomes attributed to some probiotics and functional foods confirms the importance of the microbiota, even if its capabilities and the protective mechanisms against DNA damage are not fully elucidated (Commane et al., 2005; Capurso et al., 2006; Walia et al., 2014). On the contrary, when the gut clostridia and bacteroides overnumber lactobacilli and bifidobacteria, the resulting modified microbiota is considered as a predisposing factor for neoplastic lesions (Burns and Rowland, 2004; Erba et al., 2014). Moreover, fecal microbiota can modulate genotoxic and mutagenic risks in the gut (Davis and Milner, 2009; Aune et al., 2012; Power et al., 2014).

The diet-related metabolomic features of the digestive tract may endogenously affect the induced expression of fecal microbial enzymes (β-glucuronidase, nitroreductase, azoreductase, 7-α-dehydroxylase) involved in the metabolic activation of pre-carcinogen, and the activity of mucolytic enzymes (β-glucosidase, *N*-acetyl-β-D-glucosaminidase) that increase colonocyte susceptibility to genotoxic compounds (Cenci et al., 1993; Fotiadis et al., 2008; Villarini et al., 2008). Furthermore, even secondary bile acids, nitrosamines and fecapentaenes originated by microbial metabolism have genotoxic properties (de Kok and van Maanen, 2000). The intestine may sometimes receive exogenous food genotoxins, such as mycotoxins, plant glycosides, and various additives or genotoxic compounds originated from cooking processes (i.e., polycyclic aromatic hydrocarbons and heterocyclic amines; Goldman and Shields, 2003; Naccari et al., 2009). Hence the interest in dietary habits influencing the composition of intestinal microbiota and, consequently, its modulatory activity on genotoxicity and colon cancer, is relevant (Gratz et al., 2011; Claesson et al., 2012; Sharma and Shukla, 2016).

The impact of lifestyle, in particular omnivorous, vegetarian, and vegan habits, on gut microbiota has been recently investigated (De Filippis et al., 2015; Ferrocino et al., 2015). However, the influence of these food habits on the risk factors of chronic bowel diseases was not elucidated. In fact, while meat-based diets have been associated to an increased risk for non-communicable diseases in comparison to vegetarian diets (Orlich et al., 2015), information inherent the vegan diet is still limited (Glick-Bauer and Yeh, 2014). In this respect, many studies pointed out the possibility to assess the genotoxicity of fecal water (FW) as preventive marker for evaluating the potential risk for CRC and other intestinal pathologies (Glinghammar et al., 1997; de Kok and van Maanen, 2000; Gratz et al., 2011; Erba et al., 2014).

The objective of this study was to investigate the effects of lacto-ovo-vegetarian and vegan dietary habits, compared to the omnivorous diet, on selected groups of viable and metabolically active fecal microorganisms and on FW genotoxicity and cytotoxicity, as biomarkers for the risk assessment of chronic intestinal pathologies.

## Materials and Methods

### Subjects and Diets

Twenty-nine healthy volunteers recruited in Parma (North Italy) were assembled comprising 12 lacto-ovo-vegetarians (three males and nine females, age 39 ± 10 years, body mass index (BMI) 20.7 ± 2.2 kg/m^2^), 10 vegans (seven males and three females, age 33 ± 7 years, BMI 22.3 ± 2.2 kg/m^2^), and seven omnivores (four males and three females, age 41 ± 9 years, BMI 22.6 ± 1.7 kg/m^2^). Participants were excluded according to the following criteria: vegan (V), lacto-ovo-vegetarian (L) and omnivore (O) dietary pattern followed for less than 1 year, age under 18 or over 60 years, regular consumption of drugs, regular supplementation with prebiotics or probiotics, consumption of antibiotics in the previous 3 months, evidence of intestinal pathologies (Crohn’s disease, chronic ulcerative colitis, bacterial overgrowth syndrome, constipation, celiac disease, irritable bowel syndrome), and other pathologies (type I or type II diabetes, cardiovascular or cerebrovascular diseases, cancer, neurodegenerative disease, rheumatoid arthritis, allergies), pregnancy and lactation. The V and L volunteers were recruited with the collaboration of the Italian Society of Vegetarian Nutrition (http://www.scienzavegetariana.it/). The study was approved by the Ethics Committee of Province of Parma (No. 22884) and volunteers gave their written informed consent.

Total food and beverage consumption was assessed by means of a 7-day weighed food diary, completed every day for a total of 7 days as previously described (Dall’Asta et al., 2012). Macro- and micronutrient daily intake was calculated using a Microsoft Access application linked to the Food Database of the European Institute of Oncology, covering the nutrient composition of more than 900 Italian foods (IEO, 2008). The nutrient composition of vegetarian products was evaluated on the basis of information given by suppliers or written on the package. The nutrient intake of participants is shown in **Table 1**.

**Table 1:** Daily energy and nutrient intake of the studied subjects assessed by a 7-day weight food diary.

	Omnivores	Lacto-ovo- vegetarians	Vegans
Energy (kcal/day)	2594 ± 511	2318 ± 262	2417 ± 557
Total protein (g/day)	95 ± 15	69 ± 13	66 ± 21
Vegetable protein (g/day)	35 ± 10	50 ± 10	66 ± 21
Animal protein (g/day)	60 ± 12	19 ± 11	–
Total fat (g/day)	110 ± 20	96 ± 20	82 ± 42
SFA^a^ (g/day)	42 ± 7	30 ± 12	15 ± 8
MUFA^b^ (g/day)	50 ± 12	49 ± 9	46 ± 27
PUFA^c^ (g/day)	18 ± 6	17 ± 6	21 ± 12
Total carbohydrates (g/day)	295 ± 79	305 ± 43	365 ± 74
Soluble carbohydrates (g/day)	111 ± 16	105 ± 27	179 ± 140
Fiber (g/day)	23 ± 6	34 ± 9	49 ± 14


*^a^Saturated fatty acids; ^b^Monounsaturated fatty acids; ^c^Polyunsaturated fatty acids.*

### Fecal Sample Collection

Volunteers were instructed on methods for sample collection and were provided with specimen collection kit. Fecal samples were collected at home and transferred into the sterile sampling containers (VWR, Milan, Italy) by using a polypropylene spoon (two spoons of about 10 g) and immediately stored at 4°C. The specimens were transported to the laboratory within 12 h after the collection at refrigerated temperature. Every volunteer was requested to collect feces samples over a time span of 3 weeks, once per week.

### Microbial Counts

Ten grams of stool samples were homogenized with 90 ml of Ringer’s solution (Oxoid) for 2 min in a stomacher (Seward, London, UK) at room temperature. Decimal dilutions in quarter-strength Ringer’s solution were prepared, and aliquots of 0.1 ml of the appropriate dilutions were spread on suitable selective media for the recovery of bacteria commonly found in fecal samples. Incubation was done anaerobically: at 37°C for total anaerobes, bifidobacteria, *Bacteroides. Bacteroides–Prevotella*; at 25°C for mesophilic lactobacilli. Incubation was done aerobically: at 37°C for coliforms, corynebacteria, enterobacteria, enterococci, staphylococci, streptococci-lactococci; at 25°C for *Aeromonas–Pseudomonas*. Ten colonies, from each cultured medium, were picked and observed microscopically in order to confirm the expected morphology. The media were supplied by Merck-Millipore (Darmstadt, Germany), Sigma–Aldrich (Milan, Italy), Oxoid (Milan, Italy), Becton Dickinson (Milan, Italy). Details are given in **Table 2**. Results were expressed as the mean of log_10_ colony forming units (CFU) per gram from three independent determinations.

**Table 2:** Number of viable representative bacteria found in fecal samples obtained from omnivores (*n* = 21), lacto-ovo-vegetarians (*n* = 36) and vegans (*n* = 30).

Microbial group	Omnivores	Lacto-ovo-vegetarians	Vegans	*P*-value^∗^
Total anaerobes^a^	9.45 ± 0.42^A^	9.10 ± 0.36^B^	9.49 ± 0.27^A^	0.028
**Gram-negative bacteria**				
*Bacteroides fragilis* group^b^	6.17 ± 0.52	6.20 ± 1.07	5.88 ± 1.20	0.745
*Bacteroides–Prevotella*^c^	8.33 ± 0.76	8.61 ± 0.38	8.82 ± 0.58	0.229
Coliforms^d^	6.98 ± 0.72	6.45 ± 0.92	6.44 ± 0.80	0.351
Enterobacteria^e^	7.16 ± 0.83	6.71 ± 0.76	6.54 ± 0.72	0.264
*Aeromonas–Pseudomonas*^f^	6.53 ± 0.86	6.03 ± 0.87	5.91 ± 1.12	0.408
**Gram-positive bacteria**				
Bifidobacteria^g^	8.93 ± 0.57	8.72 ± 0.79	8.01 ± 1.15	0.087
Mesophilic lactobacilli^h^	7.78 ± 0.54	7.00 ± 0.97	7.16 ± 0.75	0.146
Enterococci^i^	6.70 ± 0.69	6.39 ± 1.23	5.88 ± 0.69	0.223
Streptococci–lactococci^j^	8.52 ± 0.52	8.54 ± 0.53	8.71 ± 0.32	0.642
Staphylococci^k^	6.09 ± 0.62^A^	5.29 ± 0.85^B^	5.07 ± 0.71^B^	0.030
Corynebacteria^l^	5.45 ± 0.88^A^	4.63 ± 1.14^A,B^	4.11 ± 0.73^B^	0.034


*Mean values ± SD (log_10_ CFU/g) obtained from samplings carried out in three consecutive weeks. ^∗^One-way ANOVA. Homogeneity of variances was checked using Bartlett’s test. ^A,B^Values in a row with different superscript letters are significantly different (*p* < 0.05) according to Bonferroni’s multiple comparison post-test. Culture media: ^a^Wilkins-Chalgren Anaerobe Agar plus defibrinated blood, Oxoid; ^b^Bacteroides Bile Esculin Agar, BD; ^c^Wilkins-Chalgren Anaerobe Agar plus G-N supplement and defibrinated blood, Oxoid; ^d^Chromocult Coliform Agar, Merck; ^e^MacConkey agar N°2, Oxoid; ^f^GSP agar plus penicillin G, Sigma; ^g^Bifidobacterium Agar, BD; ^h^De Man Rogosa and Sharpe agar, Oxoid; ^i^Slanez and Bartley plus Tween 80 and sodium carbonate, Oxoid; ^j^M17 agar plus glucose, Oxoid; ^k^Mannitol Salt Agar plus egg yolk solution, Oxoid; ^l^Hoyle medium base plus laked horse blood and potassium tellurite, Oxoid.*

### Fecal Water Preparation

Samples were processed as described previously by Bianchi et al. (2010). Feces were defrosted at room temperature and diluted 1:1 with phosphate buffered saline (PBS) (Dulbecco’s 1X; Applichem GmbH, Darmstadt, Hesse, Germany) and homogenized (2 × 2 min) in a Stomacher (Seward, London, UK). The homogenates were transferred into polypropylene tubes and centrifuged (20,000×*g*, 2 h, 4°C) using a high-speed refrigerated centrifuge (3K-30; Sigma Laboratory Centrifuge, Osterode, Saxony, Germany). The supernatants, representing the FW fraction, were stored in aliquots (100–200 μl) at -80°C until further analysis.

### Fecal Water Testing for Toxicity

Cytotoxicity and genotoxicity of FW fractions were evaluated using the stabilized human adenocarcinoma HT29 colonocytes as target. HT29 cells were grown in Dulbecco’s modified Eagle’s medium (Sigma–Aldrich) containing 10% fetal bovine serum, penicillin (100 IU/ml) and streptomycin (100 IU/ml) and incubated under 5% CO_2_ at 37°C. Before performing cytotoxicity and genotoxicity tests, FW samples were co-incubated with HT29 suspension (ca. 2.0 × 10^6^ cells/ml) (1:1, V/V) at 37°C for 30 min.

### Cytotoxicity

HT29-FW suspensions were examined for cytotoxicity by the trypan blue dye exclusion assay (Freshney, 1987). Cytotoxicity was measured using a Countess (Invitrogen Srl, Milan, Italy) automated cell counter. Briefly, aliquots of cell suspensions were mixed with equal volumes of 0.4% trypan blue, with 10 μl loaded onto a Countess cell counting chamber slide. The instrument is equipped with a camera that acquires images from cell samples on the chamber slide, and the image analysis software automatically analyzes acquired cell images and measures cell count and viability. Cell viability was calculated as the percentage (%) of viable cells after FW treatment compared with untreated controls (PBS). Results were expressed as follows:

$$\begin{array}{c} \text{Viability}(\%)=[\left(\frac{\text{sample absorbance}}{\text{control absorbance}}\right)]\times 100\\ \text{Cytotoxicity}(\%)=100-\text{percent viability.} \end{array}$$

### Genotoxicity

FW induced primary DNA damage was evaluated by the standard alkaline procedure (lysis at pH 10, unwinding, and electrophoresis at pH > 13) of the single-cell microgel-electrophoresis (comet) assay (Singh et al., 1988; Tice et al., 2000) with minor modifications (Moretti et al., 2013; Dominici et al., 2014); PBS and 0.75 μM 4-nitroquinoline-1-oxide (4-NQO) were used as negative and positive controls, respectively. All the steps of the comet assay were conducted under yellow light to prevent the occurrence of additional DNA damage.

Briefly, immediately after exposure aliquots (400 μl) of HT29-FW suspension were included in 120 μl of 0.7% low melting-point agarose (Sigma–Aldrich) maintained at 37°C, and 65 μl were immediately spread onto microscope slides pre-coated with 1.0% normal melting-point agarose (Sigma–Aldrich). Subsequently, the agarose embedded cells were lysed by immersing the slides in ice-cold freshly prepared high-salt solution with detergents (10 mM Tris–HCl, 2.5 M NaCl, 100 mM Na2EDTA, 1% Triton X-100, and 10% dimethyl sulfoxide (DMSO); pH 10). After lysis, the slides were placed in an electrophoresis tank (HU25-37; Scie-Plas Ltd., Southam, UK) and left for 20 min at 4°C in the high-pH (>13) electrophoresis buffer (300 mM NaOH, 1 mM Na 4 EDTA, pH > 13) to allow the DNA to unwind. Electrophoresis was then performed, on ice, in the same buffer for 20 min at 1.0 V/cm and 300 mA. Afterward, the slides were washed twice with the neutralization buffer (0.4 M Tris-HCl, pH 7.5) and fixed in 70% ethanol for 5 min. The slides were dried and stored overnight before microscopic observation. Staining of slides was performed immediately before analysis using 65 μl of 10 μg/ml ethidium bromide (Sigma–Aldrich). For each experimental point duplicate slides were prepared, and 100 nucleoids were screened per each sample (50 comets from each slide) at 200× magnification with an epifluorescent microscope (Olympus BX41, Tokyo, Japan) under a 100 W high-pressure mercury lamp (HSH-1030-L, Ushio, Japan) using appropriate optical filters (excitation filter 510–550 nm and emission filter 590 nm). The extent of induced DNA strand breakage was measured by a computer-based semi-automated image analysis system (Comet Assay III, Perceptive Instruments, Haverhill, UK). Computerized imaging was described in detail elsewhere (Moretti et al., 2002) and was performed (blind) on coded slides. Percent of fluorescence migrated in the comet tail (i.e., tail intensity; Collins, 2004) was used to measure the level of DNA damage produced by FW incubation. Results were expressed as the mean DNA tail intensity ± standard deviation from 100 counts. Each FW sample was analyzed with three replicates.

### Statistical Analysis

All experiments were performed on triplicate and results were expressed as mean ± standard deviation. One-way analysis of variance (ANOVA) followed by Bonferroni’s multiple comparison post hoc test were used for a global comparison of microbial counts, genotoxicity and cytotoxicity in relation to the three dietary groups. Homoscedasticity was evaluated by Bartlett’s test. Unpaired Student’s *t*-test with Welch’s correction was used for a direct comparison of genotoxicity and cytotoxicity results of two dietary groups. Statistical significance was set at a two tailed *p*-value ≤ 0.05. Relation between toxicological parameters was assessed by Pearson coefficient. Analyses were done with GraphPad Prism software version 5 (La Jolla, CA, USA).

## Results

### Fecal Bacteria Composition

For each subject, fecal bacteria composition was quite stable throughout the samples collected during the three consecutive weeks. Moreover, it should also be specified that in each dietary group limited differences among subjects were found (data not shown).

As shown in **Table 2**, the mean levels of the researched bacteria were slightly different in the three groups of the examined subjects, so that the global differences among groups found by one-way ANOVA generally do not reach the statistical significance. However, some significant differences (*p* < 0.05) among dietary habits were evidenced by post hoc Bonferroni’s test for total anaerobes, staphylococci and corynebacteria, while for others microorganisms (i.e., enterobacteria, bifidobacteria, *Bacteroides–Prevotella*) appreciable, but not significant, differences among dietary groups were still evident. The average amount of total anaerobes in lacto-ovo-vegetarians was significantly lower compared to that in vegans and omnivores (*p* = 0.028). Similarly, the population counts of corynebacteria and staphylococci in the omnivore group were higher than those of the other groups (*p* < 0.05). Despite no significant differences were observed among the three groups, vegans showed lower levels of bifidobacteria and mesophilic lactobacilli. On the other hand, especially in vegans, but also in lacto-ovo-vegetarian group, higher *Bacteroides–Prevotella* levels were found compared to those observed in omnivores.

### Fecal Water Toxicity

The toxicological investigations are based on fecal samples collected in two consecutive weeks. As shown in **Table 3**, one-way ANOVA analysis emphasized for both genotoxicity and cytotoxicity more marked differences among the three dietary groups than those observed for microbial counts. In particular, although both vegetarian diets (i.e., lacto-ovo-vegetarian and vegan) reduced the genotoxicity levels in FW compared to the omnivore one, only the lacto-ovo-vegetarian one demonstrated a significant impact. Cytotoxicity decreased in samples from lacto-ovo-vegetarians, while increased in those from vegans, but differences between these groups were not statistically significant.

**Table 3:** Genotoxicity and cytotoxicity found in fecal water samples of omnivores (*n* = 14), lacto-ovo-vegetarians (*n* = 24), and vegans (*n* = 20).

Biomarker	Omnivores	Lacto-ovo-vegetarians	Vegans	*p*-value^∗^
Genotoxicity (tail intensity)^a^	2.08 ± 0.97^A^	0.92 ± 0.46^B^	1.49 ± 0.68^A^	0.014
Cytotoxicity (percent dead cells)^b^	19.86 ± 8.38^A,C^	14.25 ± 3.83^A,B^	25.70 ± 13.63^C^	0.043


*Mean values ± SD obtained from samplings carried out in two consecutive weeks. ^∗^One way ANOVA. Homogeneity of variances was checked using Bartlett’s test. ^A,B,C^Values in a row with different superscript letters are significantly different (*p* < 0.05) according to Bonferroni’s multiple comparison post-test. ^a^Comet assay, ^b^Trypan blue exclusion.*

**FIGURE 1:**
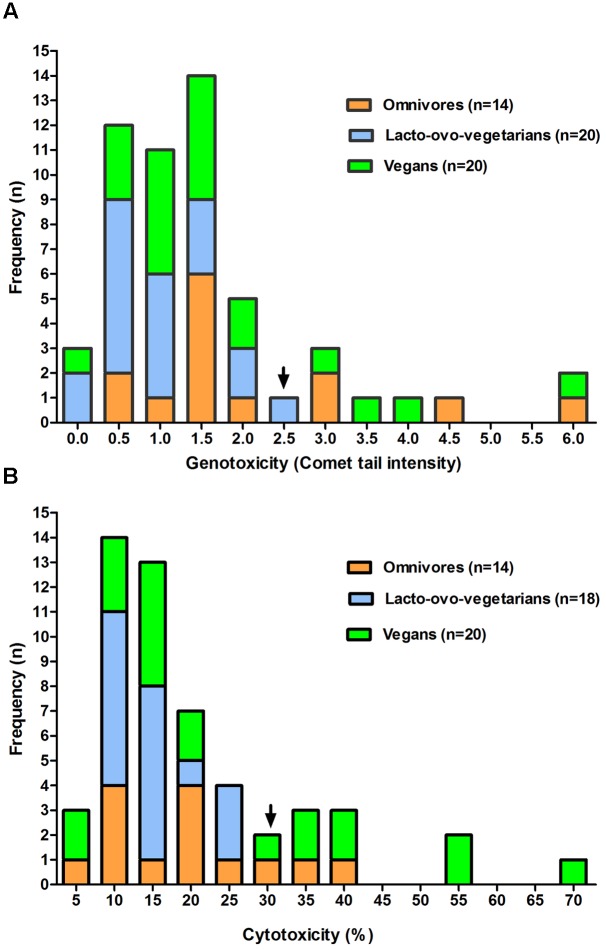
**Frequency distribution of genotoxicity** **(A)** and cytotoxicity **(B)** levels observed for fecal water samples from omnivores, lacto-ovo-vegetarians, and vegans. Only data relative to subjects showing no significant change in the samples of the 2 weeks are included. For each box the arrow indicates an approximation to normality in the first part of the figure.

The comparative analysis of the frequency levels of FW cytotoxicity and genotoxicity observed in the dietary groups shows similar behavior for both parameters considered (**Figures 1A,B**). In fact, even in the presence of a limited number of samples, an evident approximation to normality could be observed in the first portion of the genotoxicity (up to 2.5) and cytotoxicity (up to 30) frequency distribution. Interestingly, the positive skew of both distributions (right tail) included samples of omnivores and vegans, but not of lacto-ovo-vegetarians.

**FIGURE 2:**
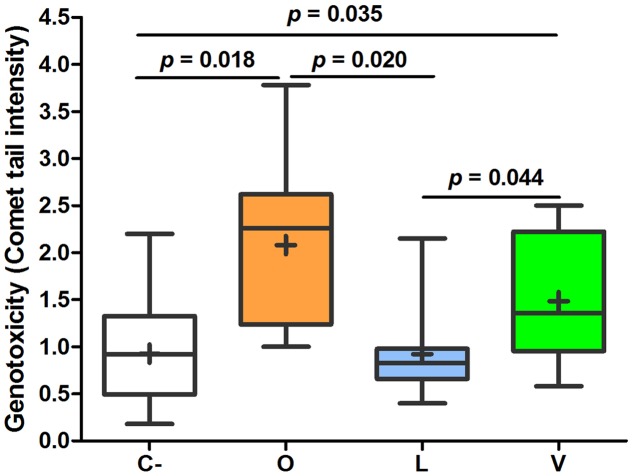
**Relative quantification of genotoxic effect in fecal waters samples of omnivores (O), lacto-ovo-vegetarians (L), vegans (V), and negative controls (C-).** Box and Whisker plots with median, 25–75th percentiles, range and mean as “+”. DNA tail intensity of positive controls (0.75 μM 4-NQO instead of FW) was 10.34 ± 2.36. The *p*-values were obtained by unpaired *t*-test.

**FIGURE 3:**
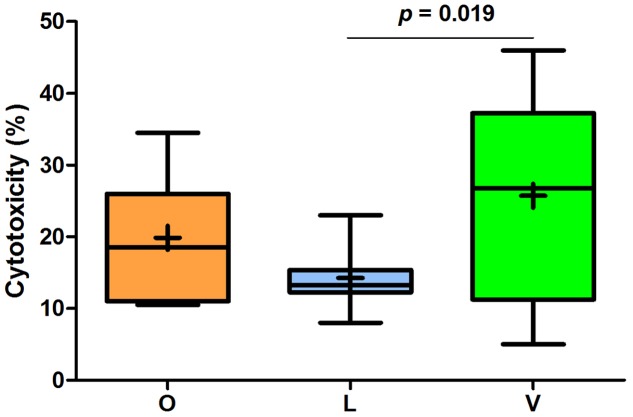
**Relative quantification of cytotoxic effect in fecal waters samples of omnivores (O), lacto-ovo-vegetarians (L), and vegans (V).** Box and Whisker plots with median, 25-75th percentiles, range and mean as “+”. The *p*-values were obtained by unpaired *t*-test.

Analyzing the mean values of FW genotoxic and cytotoxic effects, some important differences among the three groups were found. As shown in **Figure 2**, FW genotoxicity of lacto-ovo-vegetarians was significantly lower than that of omnivores and vegans. The levels found in vegans were not statistically different from those of omnivores (*p* = 0.246). Furthermore, the mean comet DNA tail intensity of lacto-ovo-vegetarians corresponded to that of negative control (*p* = 0.858). Similarly, as reported in **Figure 3**, cytotoxicity levels were significantly different between lacto-ovo-vegetarians and vegans, showing the lowest and highest values, respectively. Nonetheless, in both cases no significant differences with omnivores were found (L vs O, *p* = 0.081; V vs O, *p* = 0.896). Surprisingly, a marked variability characterized the vegan samples.

**FIGURE 4:**
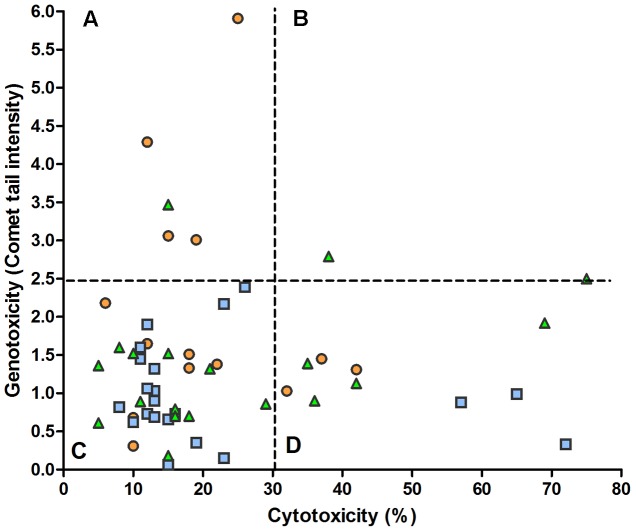
**Relation between fecal water cytotoxicity and genotoxicity.** The overall analysis of 52 samples from omnivores (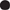), lacto-ovo-vegetarians (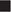), and vegans (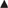) showed no significant regression (*y* = 1.34 + 0.003*x*) and correlation (*r* = 0.049). The dashed lines delimit the reference levels for the normality approximation (see **Figure 1**). **(A)** low cytotoxicity, high genotoxicity; **(B)** high cytotoxicity and genotoxicity; **(C)** low cytotoxicity and genotoxicity; **(D)** high cytotoxicity, low genotoxicity.

Examining the levels of cytotoxicity and genotoxicity found in each sample, **Figure 4** shows the absence of correlation between the two variables. In particular, it appears that a substantial part of samples (21%) characterized by appreciable cytotoxicity has low values of genotoxicity (D sector) and, otherwise, some samples (9.6%) with a low cytotoxicity present the highest values of genotoxicity observed in the present study (A sector).

## Discussion

Recent reports evidenced that the diet can influence the composition of intestinal microbiota, as reported in studies using metagenomic approaches for the characterization of the human gut microbiome (Turnbaugh et al., 2009; Ferrocino et al., 2015). However, little is currently known about the combined relationship among diet, viable gut microbiota and FW toxicology.

This study has analyzed fecal samples obtained from 29 Italian healthy volunteers who followed omnivorous, lacto-ovo-vegetarian or vegan diets and with energy and nutrient intakes aligned with their dietary pattern. The prevalence of bacterial groups considered normal inhabitants of the human intestine, together with the genotoxicity and cytotoxicity of FW, has been studied.

Considering that protective or detrimental effects of gut microbiota should necessarily be linked to their overall activity, our culture-dependent approach did not aim to the identification of the single microbial species. On the contrary, we used viable counts on different selective culture media because our main aim was to enumerate specific microbial groups commonly found in human feces. In this respect, despite it is commonly accepted that 80% of the bacterial species in the human gut cannot be cultured, a renewed interest in culture methods was recently reported (Lagier et al., 2012; Browne et al., 2016). The novelty of the present work is mainly related to the fact that, for the first time, the possible relation among the quantity of viable fecal bacteria and the levels of FW genotoxicity and cytotoxicity has been investigated in subjects with different dietary habits.

We showed that most microbial groups did not differ significantly in the three dietary habits, as also previously evidenced in a similar case study (Ferrocino et al., 2015). However, the observed differences of viable counts found for certain bacteria could suggest a particular role of the lacto-ovo-vegetarian and vegan diets in the modulation of the gut viable bacteria, compared to omnivorous diet. The values found for omnivores were compatible with those previously described (Zimmer et al., 2012; Matijasic et al., 2014).

In contrast with the main result of a recent study (Ferrocino et al., 2015), our survey showed counts of total anaerobes significantly lower in lacto-ovo-vegetarian feces than in those of omnivores and vegans. Nevertheless, in line with the above reference, no significant differences in bifidobacteria were observed among the three dietary groups, although in our case the lowest amount of these bacteria was found in vegan stool samples. Low bifidobacteria content has been associated with diets rich in amylase resistant starch (Scott et al., 2013) and found, together with low concentrations of mesophilic lactobacilli, in samples from non-omnivores when evaluating changes in fecal microflora related to host age (Mariat et al., 2009). In general, a decrease of bifidobacteria and lactobacilli would predict a decrease in the gut protection (Thomas et al., 2011), also in accordance with findings regarding probiotics (Azcarate-Peril et al., 2011), but this was not confirmed in our survey. In this regard, it should be emphasized that FW samples of all the 29 subjects showed toxicological properties when incubated with HT29 cells. The genotoxicity levels we found for omnivores were similar to those found by Mai et al. (2009), while being relatively low compared to those reported by others (Oßwald et al., 2000). This may be caused by the different cell lines used as target (HT29, Caco-2 or lymphocytes) or by the different incubation time (5–30 min) between FW and cells, which would allow cells to operate a repair of DNA damage (Gratz et al., 2011).

Even though the number of subjects involved in the present study does not allow to establish correlations among the nutritional, microbiological and toxicological variables, interesting information on relationships between diet and associated gut risk factors can be deduced. Our data clearly show that, despite the absence of substantial changes in the viable counts of the fecal bacteria, vegetarian and vegan diets can contribute in reducing the risk of DNA damage, as evaluated by Comet assay. The lacto-ovo-vegetarian habit, a less restrictive dietary pattern than the vegan one, was particularly effective in lowering the levels of both FW genotoxicity and cytotoxicity. This finding highlights the possible contribution of this dietary habit in reducing the risk of carcinogenesis at intestinal level and is consistent with our previous studies concerning the antigenotoxic activity of some probiotics and food-borne microorganisms in animal models (Villarini et al., 2008; Dominici et al., 2014). Indeed, the capacity of food-borne bacteria and yeasts with acid-bile resistance to significantly reduce the activity of model genotoxins, such as nitroarenes, alkylating agents, mycotoxins, and heterocyclic amines, has been already demonstrated *in vitro* (Caldini et al., 2005; Cenci et al., 2008). Despite in the present study the number of lactobacilli in fecal samples was not influenced by diets, we can hypothesize that the antigenotoxic properties of autochthonous lactobacilli and yeasts present in dairy foods (Corsetti et al., 2008; Trotta et al., 2012) could mediate the observed beneficial effects of the lacto-ovo-vegetarian diet. On the other hand, these benefits could as well be ascribed to the abundance of non-digestible carbohydrates and fibers in vegetarian diets, that can play an important role in reducing fecal genotoxicity (Johansson et al., 1992). Furthermore, it is well known that meat-based diets with high protein content can lead to the production of genotoxic metabolites in the intestine, such as N-nitroso compounds (Gratz et al., 2011) besides heterocyclic amines and/or amino acid pyrolysis products as a result of heating (Kassie et al., 2001).

## Conclusion

We conclude that the vegetarian diets herein considered, when compared to omnivore dietary habits, even if not showing drastic modifications of the viable fecal bacteria considered, seem able to affect the intestinal ecosystem activities related to fecal genotoxicity and cytotoxicity.

Coherently with nutritional and gastroenterological aspects inherent diet-health beneficial relationships (Sabate, 2003; Clarke et al., 2012) our findings highlight the important role of the lacto-ovo-vegetarian diet and, to a lesser extent of the vegan one, in reducing FW genotoxicity. Moreover, even considering the low levels of FW cytotoxicity found for lacto-ovo-vegetarian diet, the results of this study further support its potential role in gut health and in the protection from inflammatory bowel diseases and prevention of CRC.

## Author Contributions

GC and AC designed the study. GC, EF, and MM analyzed the data and drafted the manuscript; NP and CL enrolled subjects and helped the study design; CL and RP performed the experiments, discussed the results and contributed to the work at various stages. All authors read and approved the final manuscript.

## Conflict of Interest Statement

The authors declare that the research was conducted in the absence of any commercial or financial relationships that could be construed as a potential conflict of interest.
